# Yeast polysaccharides modulate lipid metabolism and restore oviduct inflammatory and microbial homeostasis to support egg quality in *Salmonella* Pullorum-challenged hens

**DOI:** 10.1186/s40104-026-01377-z

**Published:** 2026-04-08

**Authors:** Jianmin Zhou, Dong Dai, Yu Fu, Haijun Zhang, Guanghai Qi, Jing Wang

**Affiliations:** Key Laboratory of Feed Biotechnology, Ministry of Agriculture and Rural Affairs, Institute of Feed Research, Chinese Academy of Agricultural Sciences, Beijing, 100081 China

**Keywords:** Egg quality, Laying hen, Lipid metabolism, Oviduct microbiota, *Salmonella* Pullorum, Yeast polysaccharides

## Abstract

**Background:**

Our previous work demonstrated that yeast polysaccharides (YP) possess prebiotic and immunomodulatory activity and improve performance, immunity via modulating gut microbiota in laying hens. Building on these findings, the present study examined whether YP additionally modulate layers lipid utilization and related metabolic signatures under basal conditions and mitigates oviduct dysfunction and egg-quality deterioration during *Salmonella* Pullorum challenge.

**Methods:**

A total of 288 Hy-Line Brown hens (35 weeks) were fed diets containing 0, 250, 500, or 1,000 mg/kg YP for 12 weeks. Egg quality and apparent total tract digestibility were recorded, and untargeted plasma metabolomics was performed in CON and YP1000 hens. Thereafter, hens from the CON and YP1000 groups were orally inoculated with *S.* Pullorum or saline, generating the CON, SAL, and YP + SAL groups. Post-challenge assessments included egg quality, magnum histology, oviductal albumen antimicrobial proteins, serum and oviduct cytokines, *Salmonella* load and LPS levels, and oviduct microbiota composition.

**Results:**

Under basal conditions, YP linearly increased yolk color at week 12 (ANOVA, *P* = 0.043; linear, *P* = 0.015; quadratic, *P* = 0.045) and enhanced ether-extract digestibility (ANOVA, *P* = 0.004; linear, *P* < 0.001; quadratic, *P* < 0.001). Metabolomics identified 16 differential metabolites; YP1000 elevated multiple LysoPC/LysoPE species, choline, taurine-conjugated bile acids, riboflavin, and nicotinamide, and decreased PE(38:6) (*P* < 0.05). Following *S.* Pullorum challenge, SAL hens showed reduced albumen height and Haugh unit, magnum epithelial disruption, heightened IL-1β and TNF-α responses, increased *Salmonella* and LPS burdens, reduced ovotransferrin and lysozyme levels, and a Proteobacteria-enriched, *Lactobacillus*-depleted oviduct microbiota (*P* < 0.05). YP supplementation mitigated these outcomes: YP + SAL hens maintained internal egg quality near control values, preserved magnum structure and antimicrobial protein secretion, increased serum IL-10 while normalizing IL-1β and TNF-α, reduced *Salmonella* and LPS levels, and displayed higher oviduct microbial diversity with enrichment of *Lactobacillus* and fewer opportunistic Proteobacteria (*P* < 0.05).

**Conclusions:**

YP not only improve lipid utilization and yolk pigmentation but also enhance the resistance of the oviduct–egg axis to *S.* Pullorum through coordinated modulation of host inflammation and oviduct microbial balance, offering a practical nutritional tool to support egg quality and safety in layer production.

**Supplementary Information:**

The online version contains supplementary material available at 10.1186/s40104-026-01377-z.

## Background

Bacterial contamination of table eggs remains a major food safety concern, with *Salmonella* serovars such as Enteritidis and Pullorum able to persist in hens and contaminate eggs internally via the reproductive tract [1, 2]. Persistent colonization of the ovary and oviduct allows bacteria to enter yolk or albumen before shell deposition, creating a route for transmission to consumers [3, 4]. In addition to foodborne risks, reproductive tract infections and inflammation can impair egg quality, thereby reducing the economic value of eggs [5].

The hen oviduct is not only the site of shell and albumen formation but also a critical mucosal barrier that must balance antimicrobial defense with the maintenance of secretion and transport functions. Recent microbiome studies have begun to characterize the chicken oviduct as a structured “gut–oviduct–egg” continuum, with segment-specific communities dominated by Firmicutes, Proteobacteria, Bacteroidota, and Actinobacteriota [6, 7]. Building on this, our recent work showed that uterine and magnum microbiota in hens are tightly coupled to local barrier molecules, inflammatory status and epithelial cell-cycle/apoptosis programs, and that antibiotic-induced uterine dysbiosis aggravates pathological changes and compromises egg quality in hens [8]. Moreover, experimental modulation of the oviduct microbiota, such as intravaginal administration of *Lactobacillus johnsonii* or dietary *Lactobacillus crispatus*, can attenuate oviductal inflammation and improve barrier function in layers [9, 10]. These findings suggest that nutritional strategies which stabilize oviduct microbiota and immunity could help preserve egg quality under infectious challenges.

Yeast polysaccharides (YP), rich in β-glucans and mannans, have emerged as promising non-antibiotic feed additives in poultry. Mechanistic and applied studies have indicated that yeast-derived products can enhance growth performance, modulate gut morphology and tight junctions, and shift intestinal microbiota toward beneficial communities [11–13]. Similarly, our previous study in laying hens demonstrated that dietary YP linearly improved feed efficiency and egg production, alleviated LPS-induced systemic and intestinal inflammation, and promoted gut microbial stability [14]. However, whether such prebiotic and immunomodulatory effects extend to host metabolism under basal conditions and to the reproductive tract during pathogen challenge remains unclear.

Lipid handling is vital for egg formation, as yolk lipids and carotenoids are supplied via intestinal absorption and hepatic metabolism and then deposited into developing follicles [15–17]. Dietary interventions that improve lipid utilization can modify yolk composition and pigmentation, with implications for both nutritional value and consumer acceptance [18, 19]. However, little is known about whether YP influence lipid digestion and metabolism in layers. Likewise, the potential for YP to protect oviduct structure and function during *Salmonella* infection remains largely unexplored, particularly for *S.* Pullorum, an oviduct-tropic serovar of continuing relevance in some regions [2, 20].

Therefore, the present study was designed as a continuation of our previous work on yeast polysaccharides in laying hens [14]. First, we hypothesize that dietary YP supplementation would improve performance related to lipid utilization under basal conditions. For this, we examined the effects of graded dietary YP supplementation on egg quality, apparent nutrient digestibility, and plasma metabolomic profiles, with a focus on lipid-related metabolites. Second, we hypothesize that prior YP supplementation could attenuate *S.* Pullorum–induced impairments in egg internal quality and oviduct integrity. This was evaluated by determining egg internal quality, magnum morphology, immune responses, pathogen burdens, and oviduct microbiota composition. By integrating these multilevel endpoints, this study aimed to determine whether YP can be exploited as a nutritional strategy that couples improved lipid utilization with enhanced reproductive tract resilience and egg safety in laying hens.

## Materials and methods

### Birds and experimental design

A total of 288 Hy-Line Brown laying hens (35 weeks old) were randomly allocated to four dietary treatments supplemented with 0, 250, 500, or 1,000 mg/kg YP. Each treatment included six replicates of 12 birds. YP were obtained from a commercial supplier (China) and previously characterized in our laboratory, and the inclusion levels were selected based on our previous study, which demonstrated that YP supplementation within this range linearly improved performance and immune status [14]. The yeast polysaccharides mainly comprise β-glucans and mannoproteins, and acid hydrolysis revealed mannose and glucose as the predominant monosaccharides (48.83% and 47.59% of total released sugars, respectively); most fractions exhibited Mw < 100 kDa (90.75%). Before the formal trial, the hens underwent a 7-d adaptation period. Hens were housed in three-tier battery cages (three hens per cage; cage dimensions: 40 cm × 40 cm × 35 cm) under a 16-h light regimen at an intensity of 20 lx. Ambient temperature was maintained between 22 and 26 °C throughout the experiment. Feed and water were provided ad libitum in mash form and via nipple drinkers, respectively. The basal diet was formulated according to the Chinese Feeding Standard of Chickens (NY/T33-2004) [21] and is shown in Table 1. All hens remained in good health throughout the feeding period.

**Table 1:** Composition and nutrient levels of experimental diet (as-fed basis), %

Item	Content, %
Ingredient	
Corn	59.00
Wheat	10.00
Soybean meal (44.8% CP)	10.17
Cottonseed meal (60% CP)	9.00
Wheat bran	1.15
Salt	0.30
Dicalcium phosphate	0.90
Calcium carbonate	8.90
DL-Methionine (99%)	0.12
Lysine·HCl (78%)	0.13
L-Threonine (98%)	0.05
Choline chloride (50%)	0.10
Premix^1^	0.13
Phytase	0.03
Xylanase	0.02
Total	100
Nutrient level^2^	
Metabolizable energy, kcal/kg	2,690
Crude protein	16.49 (16.25)
Nonphytate phosphorus	0.33
Calcium	3.50 (4.013)
Lysine	0.75
Methionine	0.36
Methionine + cysteine	0.65
Threonine	0.55

^1^Premix supplied per kilogram of diet: vitamin A, 12,500 IU; vitamin D_3_, 4,125 IU; vitamin E, 15 IU; vitamin K_3_, 2 mg; thiamine, 1 mg; riboflavin, 8.5 mg; pyridoxine, 8 mg; vitamin B_12_, 0.04 mg; biotin, 0.1 mg; folic acid, 1.25 mg; Ca-pantothenate, 50 mg; niacin, 32.5 mg; Cu, 8 mg; Zn, 80 mg; Fe, 40 mg; Mn, 90 mg; Se, 0.3 mg; I, 1.2 mg

^2^The values in parenthesis indicate analyzed values. Others are calculated values

### *Salmonella* challenge and sampling

Following the 12-week feeding trial (dietary adaptation period), 20 hens from each of the control and YP1000 groups were randomly selected for the *Salmonella* challenge, forming the SAL and YP + SAL groups, respectively. The *Salmonella* challenge was performed according to the protocol established in our previous study [22]. Briefly, to induce a consistent infection pressure, hens in the challenge groups were orally inoculated with 1 mL of *Salmonella* Pullorum (CVCC521, 1 × 10^9^ CFU/mL) once daily for three consecutive days using a sterilized crop gavage needle. Meanwhile, the control hens received an equivalent volume of sterile saline as the negative control (CON). Sampling was conducted at two critical stages to monitor infection dynamics: the acute phase (2–3 d post-challenge) to assess the initial immune activation and colonization [23], and the resolution phase (13–14 d post-challenge) to evaluate the re-establishment of immune homeostasis [23, 24].

Eggs were collected at weeks 6 and 12 of the feeding period to assess the effects under basal conditions, and at 2–3 and 13–14 d post-challenge to evaluate changes in egg quality and *Salmonella* load in response to infection. Before the challenge, blood was collected from the wing vein into EDTA-treated tubes, centrifuged at 1,300 × *g* for 10 min at 4 °C to obtain plasma, snap-frozen in liquid nitrogen, and stored at −80 °C for metabolomics analysis. At 3 and 14 d post-challenge, blood was again collected from the wing vein, allowed to clot, and centrifuged at 1,300 × *g* for 15 min to harvest serum, which was stored at −20 °C until analysis. Subsequently, eight hens per group at each sampling point were randomly selected, slaughtered, and dissected aseptically. The magnum was selected to investigate local host–pathogen interactions, given its critical role in limiting vertical transmission and the observed impairment in albumen quality. Approximately 2-cm segments from the middle portion of the magnum were excised, rinsed with PBS, and fixed in 10% neutral-buffered formalin for morphological examination. Ileal and magnum mucosa were aseptically scraped using sterile glass slides and immediately snap-frozen in liquid nitrogen. Microbial samples were collected by swabbing the inner surface of the magnum with sterile swabs, which were suspended in PBS in sterile cryovials. All mucosal and swab samples were stored at −80 °C until further analysis.

### Egg quality

Eggshell color (L*, a*, b* values) was measured at the equator using a colorimeter (NH310; 3nh Shenzhen, China). Eggshell thickness at the blunt end, tip, and equator, as well as eggshell strength, were determined using an Eggshell Thickness Gauge and an Egg Force Reader (ORKA Technology Ltd., Ramat HaSharon, Israel), respectively. Albumen height, Haugh unit, and yolk color were assessed using an Egg Analyzer™ (ORKA Food Technology Ltd., Ramat HaSharon, Israel).

### Apparent total tract digestibility of nutrients

To determine the apparent total tract digestibility (ATTD) of gross energy, total nitrogen, ether extract, and dry matter, 3 hens were randomly selected from each replicate and transferred to individual cages equipped with excreta collection trays at the end of the feeding period. Titanium dioxide (TiO_2_) was added to the diets at a concentration of 0.3% as an indigestible external marker for 3 d prior to the start of excreta collection. Excreta were collected twice daily at 12-h intervals for three consecutive days and stored in sealed bags at −20 °C. Any residual feed or feathers in the trays were carefully removed. Excreta collected from each cage over the 3-d period were pooled to represent one replicate, resulting in six samples per treatment. Before chemical analysis, samples were thawed, dried at 70 °C for 72 h, and finely ground to pass through a 0.5-mm screen. ATTD was calculated using the following equation:

$$ATTD=1-\left[\left({Ti}_{D}/{Ti}_{E}\right)\times \left({NUTR}_{E}/{NUTR}_{D}\right)\right]$$

where Ti_D_ and Ti_E_ (g/kg DM) are the contents of element titanium in the diet and excreta, respectively, and NUTR_D_ and NUTR_E_ (g/kg DM) are nutrient concentrations in diet and excreta, respectively.

### Plasma metabolome analysis

Plasma samples were thawed on ice, and 0.15 g aliquots were extracted with 1.5 mL of ice-cold ethanol–water (4:1, v/v). After 40 min of non-contact ultrasonication and centrifugation (16,000 × *g*, 15 min, 4 °C), the supernatants were filtered through a 0.22-µm membrane for UHPLC–HRMS analysis. A pooled quality control (QC) sample was prepared by combining 30 µL from each extract, and four solvent blanks were included to monitor background signals. Metabolite profiling was performed on a Dionex UltiMate 3000 UHPLC system coupled to a Q Exactive mass spectrometer (Thermo Fisher Scientific) using an electrospray ionization source with positive/negative polarity switching. QC samples were injected throughout the sequence to track analytical stability.

Raw data were processed using Compound Discoverer 2.1 (Thermo Fisher Scientific) for peak detection, alignment, and feature grouping. Features were extracted using a 5-ppm mass tolerance, 0.3-min retention time shift, signal-to-noise ratio of 5, and a minimum peak intensity of 2 × 10^6^. To ensure robustness, only features with QC coverage > 50%, QC relative standard deviation < 30%, and peak area > 1 × 10^5^ were retained. Metabolites were annotated against mzCloud and ChemSpider (HMDB, KEGG, LipidMAPS) using a 5-ppm mass tolerance. Putative identifications were confirmed by MS/MS spectra and retention times of authentic standards.

Multivariate analyses, including principal component analysis (PCA) and orthogonal projections to latent structures discriminant analysis (OPLS-DA) were conducted in SIMCA 18.0 (Umetrics, Umea, Sweden) after Pareto scaling and logarithmic transformation. Model validity was assessed by 200-iteration permutation tests. S-plots and variable importance in projection (VIP) values were used to identify discriminant metabolites, and statistical significance was evaluated by Student’s *t*-test. Key differential metabolites were defined as those with fold change > 1.5 or < 0.67, *P* < 0.05, VIP > 1.0, and absolute p(corr)[1] > 0.5.

### Oviduct (magnum) morphology

Samples were washed, dehydrated, cleared, and embedded in paraffin. Serial paraffin sections (5 µm) were mounted on slides, deparaffinized, rehydrated, stained with hematoxylin and eosin, sealed with neutral balsam, and examined under a light microscope (BX51; Olympus, Tokyo, Japan). Morphometric measurements were performed using ImageJ software (v1.53). The luminal epithelium height was measured at 6 randomly selected points along the mucosal folds. Glandular density was determined using a point-counting stereological method. The average value of each section was used for statistical analysis.

### Enzyme-linked immunosorbent assay

Oviduct tissues were homogenized in ice-cold phosphate-buffered saline (PBS) and centrifuged at 1,300 × *g* for 20 min at 4 °C to obtain the supernatant. Total protein in the oviduct supernatants was quantified using a bicinchoninic acid (BCA) assay kit (Nanjing Jiancheng Bioengineering Institute, Nanjing, China). The concentrations of interleukin-1β (IL-1β), interleukin-6 (IL-6), interleukin-10 (IL-10), tumor necrosis factor-α (TNF-α), lipopolysaccharide (LPS), ovalbumin, ovotransferrin, and lysozyme in serum and/or oviduct supernatants were measured using chicken-specific ELISA kits (Shanghai Enzyme-linked Biotechnology Co., Ltd., Shanghai, China), following the manufacturer’s instructions. The sensitivities of the ELISA kits were 0.1 pg/mL for IL-6, IL-10, and TNF-α, 1.0 pg/mL for IL-1β, and 0.1 EU/mL for LPS. For egg proteins, the sensitivities were 0.1 µg/mL (ovalbumin), 0.1 g/L (ovotransferrin), and 1.0 ng/mL (lysozyme). All intra- and inter-assay CVs were less than 10% and 15%, respectively.

### Quantification of *Salmonella* load

*Salmonella* load in the ileal mucosa, oviduct mucosa, and whole egg contents was quantified by absolute qPCR targeting a conserved region of the *Salmonella* 16S rRNA gene (forward: 5′-CGG GCC TCT TGC CAT CAG GTG-3′; reverse: 5′-CAC ATC CGA CTT GAC AGA CCG-3′). A standard curve was generated using ten-fold serial dilutions (10^2^ to 10^8^ copies per reaction) of a Sanger-verified recombinant plasmid containing the *Salmonella*-derived 16S rRNA amplicon. All qPCR reactions were performed in triplicate using SYBR Green chemistry on an ABI 7300 Real-Time PCR System (Applied Biosystems, Foster City, CA, USA). The *Salmonella* DNA copy number was calculated based on the standard curve and expressed as copies/g of sample.

### DNA extraction and 16S rRNA sequencing of oviduct microbiota

Microbial DNA was extracted using the E.Z.N.A.^®^ Soil DNA Kit (Omega Bio-Tek, Norcross, GA, USA) according to the manufacturer’s instructions. DNA integrity was evaluated by 1% agarose gel electrophoresis and quantified with a NanoDrop 2000 spectrophotometer (Thermo Scientific, Wilmington, DE, USA). The bacterial 16S rRNA V4 region was amplified using primers 515F (5′-GTG CCA GCM GCC GCG GTA A-3′) and 806R (5′-GGA CTA CHV GGG TWT CTA AT-3′). PCR reactions were performed on an ABI GeneAmp® 9700 thermocycler (Applied Biosystems, CA, USA) with the following conditions: 95 °C for 3 min; 27 cycles of 95 °C for 30 s, 55 °C for 30 s, and 72 °C for 45 s; and a final extension at 72 °C for 10 min. Amplicons were purified using the AxyPrep DNA Gel Extraction Kit (Axygen Biosciences, Union City, CA, USA), pooled in equimolar concentrations, and subjected to paired-end sequencing (PE300) on the Illumina MiSeq platform (Illumina, San Diego, CA, USA) following standard procedures.

After demultiplexing and quality filtering of the raw fastq files in QIIME 2, amplicon sequence variants (ASVs) were generated using the DADA2 plugin. To ensure high data quality, raw reads were filtered using standard parameters: reads were truncated at 260 bp based on quality scores, and sequences with a maximum expected error (maxEE) > 2 were discarded. Chimeric sequences were identified and removed using the consensus method. To minimize sampling bias, all samples were rarefied to a normalized depth of 30,000 reads per sample, based on the sample with the lowest valid sequence count. All sequencing was performed in a single batch on the Illumina MiSeq platform to avoid potential batch effects. Taxonomy was assigned against the SILVA (v.138) database with the classify-sklearn classifier at a confidence threshold of 0.7. Microbial data analysis was conducted on the Majorbio Cloud Platform (www.majorbio.com) of Shanghai Majorbio Bio-pharm Technology Co., Ltd. The α-diversity was assessed by the Shannon index, while β-diversity was visualized via principal coordinate analysis (PCoA) based on Bray–Curtis distances. Significant differences in community structure among treatments were determined by analysis of similarity. Furthermore, differentially abundant taxa across groups were identified through linear discriminant analysis effect size (LEfSe), employing an all-against-all comparison strategy with a linear discriminant analysis (LDA) score threshold of 4.0.

### Statistical analysis

Statistical analyses were conducted using SAS v9.2 (SAS Institute Inc., Cary, NC, USA). Data were first evaluated for normality and homogeneity of variance. One-way analysis of variance (ANOVA) followed by Tukey’s multiple range test was used to assess group differences, and linear and quadratic effects were examined by regression analysis. Differences were considered statistically significant at *P* < 0.05. Results are expressed as means ± pooled SEM. Spearman’s rank correlation analysis was performed to evaluate the associations between biological signatures and phenotypic traits within their respective experimental contexts. Specifically, correlations were assessed between differential plasma metabolites and lipid metabolism-related indices (yolk color and ether extract digestibility), as well as between differential oviductal bacterial genera and infection-related parameters.

## Results

### Effects of YP under basal conditions

#### Egg quality during feeding period

As shown in Table 2, by week 6, dietary YP supplementation had no effect on eggshell color parameters (L*, a*, b*), eggshell strength, eggshell thickness, albumen height, yolk color, or Haugh unit (*P* > 0.05). However, by week 12, a linear decrease in the eggshell b* value was detected with increasing YP levels (*P* = 0.040). In contrast, yolk color increased in a dose-dependent manner (ANOVA, *P* = 0.043; linear, *P* = 0.015; quadratic, *P* = 0.045), and hens receiving 1,000 mg/kg YP exhibited significantly higher yolk color scores than the control (*P* < 0.05).

**Table 2:** Effect of dietary yeast polysaccharides on egg quality of laying hens^1^

Item^2^	Eggshell color			Eggshell strength, N	Eggshell thickness, mm	Albumen height, mm	Yolk color	Haugh unit
	L*	a*	b*					
Week 6								
Treatments								
Control	62.78	21.08	26.54	38.52	0.45	5.25	5.08	69.05
YP250	62.65	21.21	27.01	37.65	0.45	5.59	5.03	72.87
YP500	62.66	20.94	26.38	40.36	0.48	5.49	5.28	72.22
YP1000	63.83	20.97	26.74	38.52	0.45	5.38	5.14	70.06
SEM	0.268	0.160	0.157	0.532	0.003	0.076	0.068	0.652
*P* value								
ANOVA	0.313	0.955	0.698	0.396	0.078	0.490	0.750	0.184
Linear	0.112	0.720	0.962	0.747	0.810	0.863	0.666	0.964
Quadratic	0.163	0.939	0.996	0.698	0.161	0.424	0.803	0.126
Week 12								
Treatments								
Control	62.01	21.02	27.03	42.46	0.50	6.77	5.20^b^	80.93
YP250	62.79	20.89	26.66	40.10	0.48	6.99	5.19^b^	82.99
YP500	62.76	20.56	26.60	43.18	0.49	7.03	5.64^ab^	83.34
YP1000	62.25	20.86	26.24	40.01	0.49	7.12	5.66^a^	83.88
SEM	0.261	0.154	0.129	0.584	0.003	0.104	0.084	0.697
*P* value								
ANOVA	0.720	0.695	0.229	0.237	0.630	0.795	0.043	0.636
Linear	0.927	0.657	0.040	0.386	0.542	0.350	0.015	0.251
Quadratic	0.533	0.557	0.121	0.614	0.816	0.604	0.045	0.434

^a,b^Within a column, means with no common letters differ significantly (*P* < 0.05)

^1^Means were calculated using 6 replicates (6 eggs/replicate) per treatment

^2^YP250, YP500 or YP1000: The diets supplemented with 250, 500 or 1,000 mg/kg yeast polysaccharides

#### Apparent total tract digestibility of nutrients

As shown in Table 3, dietary YP supplementation did not influence the apparent digestibility of gross energy, nitrogen, or dry matter (*P* > 0.05). However, ether extract digestibility was significantly enhanced by YP inclusion (*P* = 0.004), exhibiting linear (*P* < 0.001) and quadratic (*P* = 0.001) dose–response effects. Hens fed 500 or 1,000 mg/kg YP showed higher ether extract digestibility than the control (*P* < 0.05), with the highest value recorded in the YP1000 group.

**Table 3:** Effect of dietary yeast polysaccharides on apparent total tract digestibility of laying hens^1^

Item^2^	Experimental treatment				SEM	*P* value		
	Control	YP250	YP500	YP1000		ANOVA	Linear	Quadratic
Gross energy	0.77	0.78	0.77	0.79	0.004	0.495	0.774	0.778
Nitrogen	0.50	0.47	0.50	0.51	0.013	0.835	0.486	0.491
Ether extract	0.65^b^	0.71^ab^	0.75^a^	0.79^a^	0.017	0.004	< 0.001	0.001
Dry matter	0.70	0.71	0.71	0.72	0.005	0.856	0.479	0.697

^a,b^Within a row, means with no common letters differ significantly (*P* < 0.05)

^1^Means were calculated using 6 replicates per treatment with one pooled sample from 3 laying hens each replicate

^2^YP250, YP500 or YP1000: The diets supplemented with 250, 500 or 1,000 mg/kg yeast polysaccharides

#### Plasma metabolic signatures

**Fig. 1:**
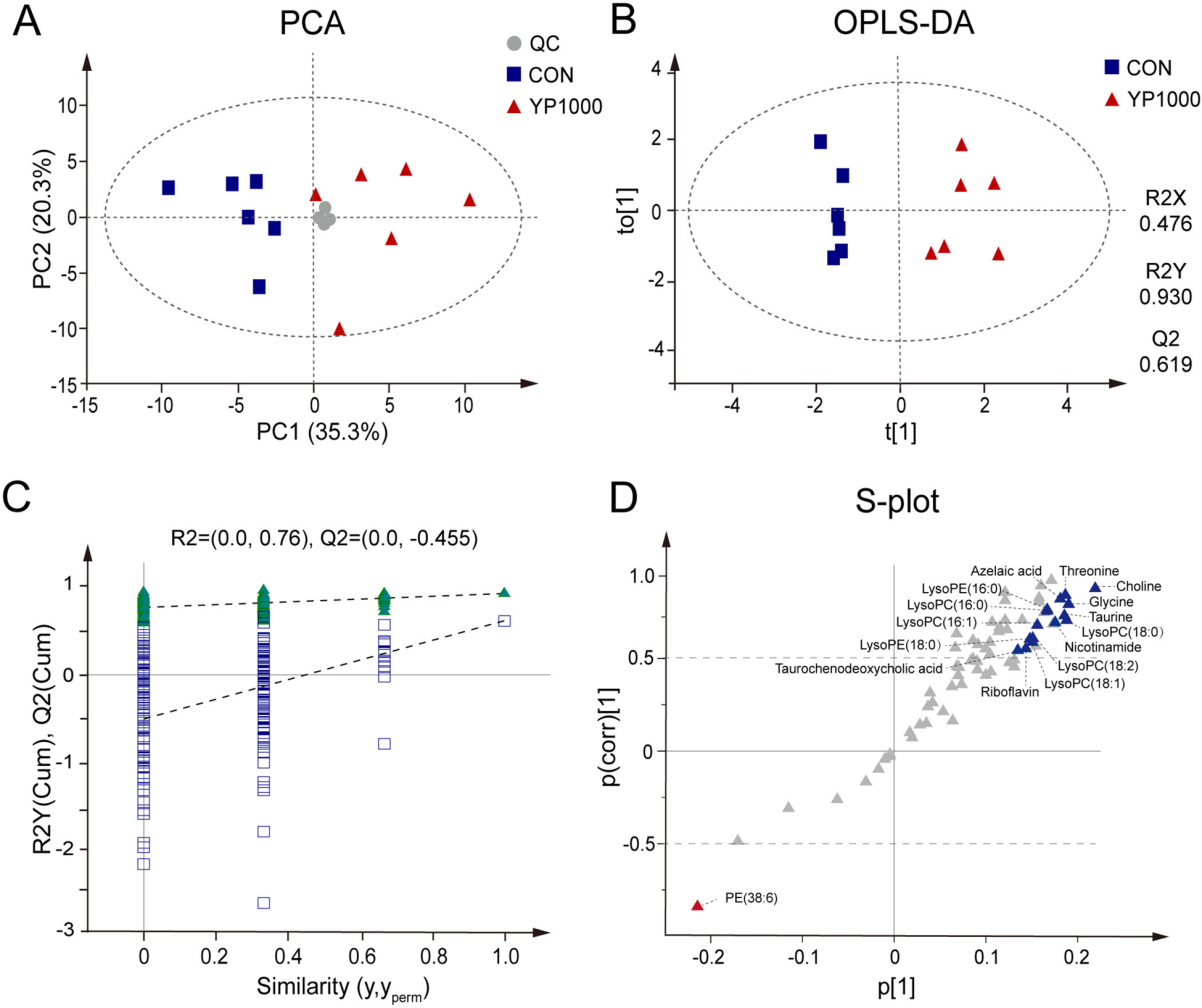
Plasma metabolomic profiling of CON and YP1000 groups (*n* = 6). **A** Principal component analysis score plot of plasma samples, where blue squares represent CON, red triangles represent YP1000, and grey circles represent QC samples. **B** Orthogonal projections to latent structures discriminant analysis (OPLS-DA) score plot showing sample distributions of the CON (blue squares) and YP1000 (red triangles) groups. **C** Permutation test plot (200 permutations) corresponding to the OPLS-DA model. **D** S-plot derived from the OPLS-DA model based on identified plasma metabolites. Colored triangles (blue/red) denote metabolites that were highlighted because they met all predefined selection criteria: |p(corr)[1]| > 0.5, VIP > 1.0, *P* < 0.05, and FC > 1.5 or < 0.67. CON, Hens received a basal diet; YP1000, Hens received a basal diet supplemented with 1,000 mg/kg yeast polysaccharides

Untargeted plasma metabolomics identified 69 metabolites that satisfied all the predefined quality-control and annotation criteria (Table S1), and these metabolites were retained for subsequent multivariate analysis. PCA revealed a clear separation trend between the CON and YP1000 groups, with QC samples clustering tightly (Fig. 1A). To further resolve group discrimination, an OPLS-DA model was constructed, which distinctly separated CON and YP1000 hens along the first predictive component (Fig. 1B). The model showed satisfactory goodness-of-fit and predictive ability (*R*^*2*^*X* = 0.476; *R*^*2*^*Y* = 0.930; *Q*^*2*^ = 0.619) and yielded a low *Q*^*2*^ intercept (−0.455), with all permuted *Q*^*2*^ values lower than the original, indicating a minimal risk of overfitting (Fig. 1C). The S-plot derived from the OPLS-DA model highlighted metabolites that fulfilled the combined selection thresholds of |p(corr)[1]| > 0.5, VIP > 1.0, *P* < 0.05, and fold change > 1.5 or < 0.67 (Fig. 1D). According to these criteria, 16 key differential metabolites were identified between the two groups (Table 4). Compared with the control, the YP1000 group showed elevated plasma concentrations of azelaic acid, choline, glycine, a range of LysoPC species, LysoPE(16:0), LysoPE(18:0), nicotinamide, riboflavin, taurine, taurochenodeoxycholic acid, and threonine, whereas PE(38:6) was reduced.

**Table 4:** Summary of key differential metabolites in laying hen plasma between Control and YP1000 groups

Putative metabolite	Retention time, min	*m/z*	Molecular formula	Fold change (YP1000/Control)	*P* value^1^	VIP^2^	p(corr)[1]^3^
Azelaic acid	5.142	188.105	C_9_H_16_O_4_	1.599	0.007	1.505	0.816
Choline	6.313	103.100	C_5_H_13_NO	1.863	0.002	1.801	0.864
Glycine	8.026	75.032	C_2_H_5_NO_2_	1.653	0.017	1.577	0.780
LysoPC(16:0)	8.293	495.332	C_24_H_50_NO_7_P	1.606	0.006	1.376	0.754
LysoPC(16:1)	7.813	493.316	C_20_H_44_N_7_O_5_P	1.612	0.006	1.284	0.674
LysoPC(18:0)	9.066	523.363	C_26_H_54_NO_7_P	1.934	0.046	1.534	0.701
LysoPC(18:1)	8.495	521.348	C_26_H_52_NO_7_P	1.676	0.043	1.226	0.587
LysoPC(18:2)	8.077	519.332	C_26_H_50_NO_7_P	1.571	0.047	1.232	0.595
LysoPE(16:0)	7.889	453.285	C_21_H_44_NO_7_P	1.525	0.001	1.375	0.761
LysoPE(18:0)	8.466	481.317	C_24_H_44_N_5_O_3_P	1.582	0.046	1.211	0.597
Nicotinamide	2.070	122.048	C_6_H_6_N_2_O	1.685	0.012	1.418	0.686
PE(38:6)	12.983	763.515	C_43_H_74_NO_8_P	0.522	0.001	1.764	−0.848
Riboflavin	6.746	376.138	C_17_H_20_N_4_O_6_	1.591	0.043	1.167	0.533
Taurine	7.721	125.014	C_2_H_7_NO_3_S	1.682	0.038	1.517	0.718
Taurochenodeoxycholic acid	6.728	499.297	C_26_H_45_NO_6_S	1.639	0.039	1.092	0.533
Threonine	8.021	119.058	C_4_H_9_NO_3_	1.562	0.011	1.517	0.825

^1^*P* values were calculated using Student’s *t*-test

^2^*VIP* Variable importance in projection, was extracted from the OPLS-DA model

^3^p(corr)[1], the loading correlation coefficient of component 1 from OPLS-DA

Metabolites were considered significantly different when VIP > 1.0, *P* < 0.05, FC > 1.5 or < 0.67, and |p(corr)[1]| > 0.5

**Fig. 2:**
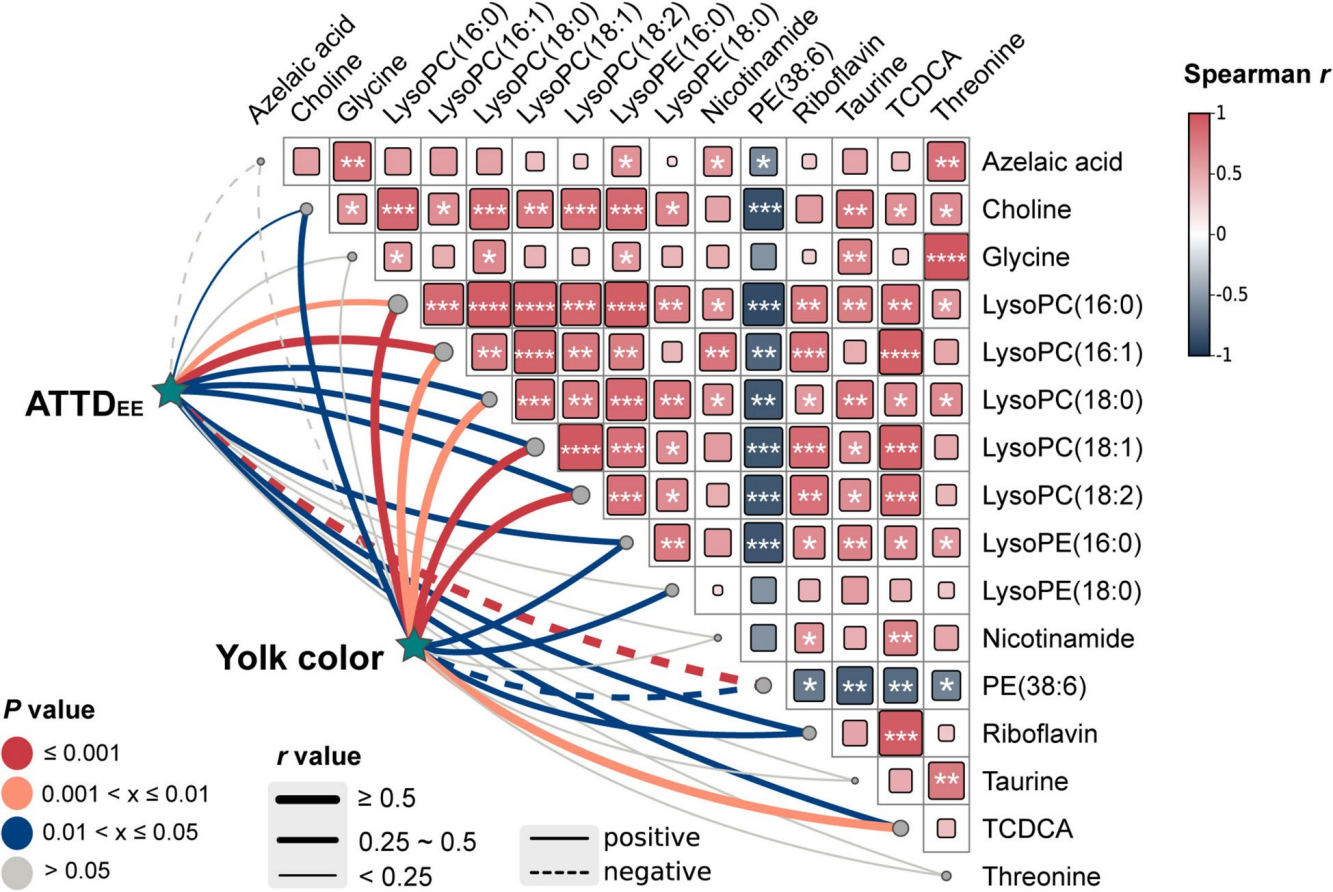
Correlation analysis between differential plasma metabolites and key phenotypic traits (ATTD_EE_ and yolk color). The triangular heatmap displays the pairwise Spearman correlations among the 16 identified differential metabolites. The color gradient represents the correlation coefficient, with red indicating positive and blue indicating negative correlations. Asterisks denote statistical significance (^*^P < 0.05, ^**^P < 0.01, ^***^P < 0.001). The connecting lines represent Spearman correlations between individual metabolites and the two host parameters. Line color indicates the statistical significance (Red: *P* ≤ 0.001; Orange: 0.001 < *P* ≤ 0.01; Blue: 0.01 < *P* ≤ 0.05). Line width is proportional to the correlation coefficient, and line type indicates the direction of the correlation (solid lines: positive; dashed lines: negative)

To further explore the functional implications of these metabolic shifts, Spearman correlation analysis was performed (Fig. 2). The results revealed that ether extract digestibility and yolk color were positively correlated with the abundance of specific lipid-related metabolites, including choline, LysoPC species (16:0, 16:1, 18:0, 18:1, 18:2), LysoPE(16:0), and taurochenodeoxycholic acid (*P* < 0.05; Fig. 2). Conversely, PE(38:6) showed significant negative correlations with both phenotypic traits (*P* < 0.05).

### Protective effects of YP under *Salmonella* challenge

#### Egg quality post Salmonella challenge

As shown in Table 5, at 2–3 d post-challenge, *Salmonella* inoculation reduced eggshell thickness and albumen height, with both SAL and YP + SAL hens showing lower values than the unchallenged control (*P* < 0.05). By 13–14 d post-challenge, differences were primarily detected in internal quality parameters. Albumen height and Haugh unit were lower in SAL hens than in CON (*P* < 0.05), whereas YP + SAL hens showed higher albumen height and Haugh unit than SAL, approaching control levels (*P* < 0.05). Eggshell traits and yolk color did not differ among treatments at this stage (*P* > 0.05).

**Table 5:** Effect of dietary yeast polysaccharides on egg quality of laying hens challenged with *Salmonella*^1^

Item^2^	Treatment			SEM	*P* value
	CON	SAL	YP + SAL		
2–3 d post-challenge					
Eggshell thickness, 10^–2^ mm	43.40^a^	39.43^b^	38.63^b^	0.715	0.009
Eggshell strength, N	37.33	31.82	29.57	1.701	0.170
Yolk color	6.25	6.75	5.63	0.266	0.221
Albumen height, mm	7.79^a^	6.77^b^	6.77^b^	0.191	0.034
Haugh unit	88.26	82.61	82.39	1.242	0.086
13–14 d post-challenge					
Eggshell thickness, 10^–2^ mm	46.17	43.58	45.88	0.784	0.345
Eggshell strength, N	39.98	36.37	41.10	1.153	0.222
Yolk color	5.75	5.63	5.75	0.092	0.837
Albumen height, mm	7.36^a^	5.57^b^	6.77^a^	0.243	0.005
Haugh unit	85.83^a^	73.89^b^	81.49^a^	1.591	0.004

^a,b^Within a row, means with no common letters differ significantly (*P* < 0.05)

^1^Means were calculated from 6 replicates (2 eggs/replicate) per treatment

^2^CON, Unchallenged control (saline gavage); SAL, *Salmonella*-challenged group; YP + SAL, Yeast polysaccharides supplementation (1,000 mg/kg) with *Salmonella* challenge

#### Oviduct (magnum) morphology

**Fig. 3:**
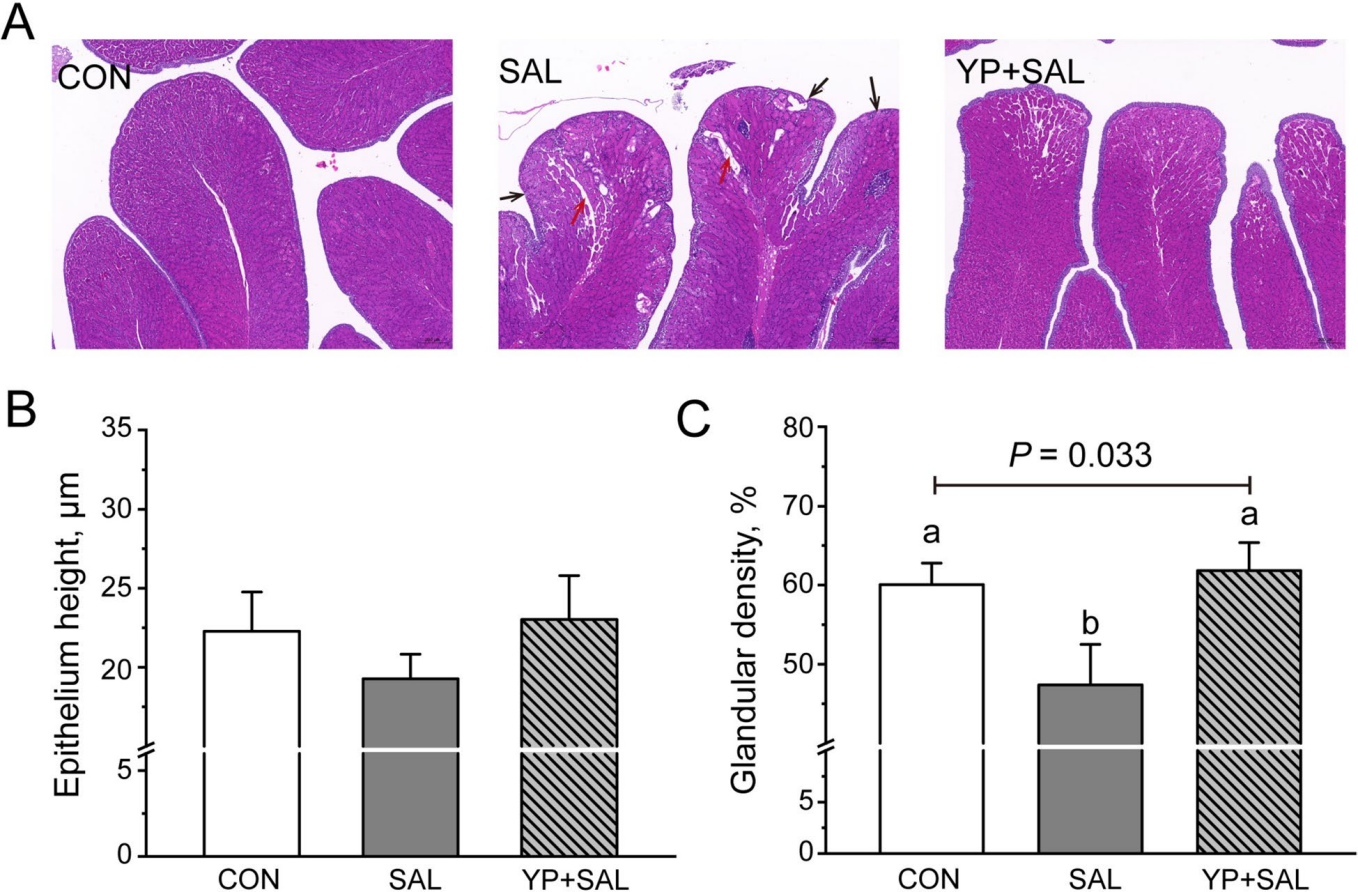
Effect of dietary yeast polysaccharides on oviduct morphology of laying hens challenged with *Salmonella*. **A** Representative histological images of the magnum (Hematoxylin and eosin (H&E) staining, 40 × magnification). Black arrows indicate epithelial discontinuities and erosion; red arrows indicate prominent luminal spaces and glandular atrophy. **B** Luminal epithelium height. **C** Glandular density. Data are presented as means ± SEM. Bars with no common letters differ significantly (*P* < 0.05). CON, Unchallenged control (saline gavage); SAL, *Salmonella*-challenged group; YP + SAL, Yeast polysaccharides supplementation (1,000 mg/kg) with *Salmonella* challenge

Morphological evaluation (Fig. 3A) revealed that *Salmonella* challenge disrupted mucosal architecture, characterized by epithelial erosion and widened luminal spaces. This damage was quantitatively confirmed by a significant reduction in glandular density in the SAL group compared with controls (*P* < 0.05; Fig. 3B and C). Conversely, YP supplementation preserved mucosal integrity and effectively reversed the decline in glandular density (*P* < 0.05).

#### Oviduct mucosal ovalbumin, ovotransferrin, and lysozyme

**Fig. 4:**
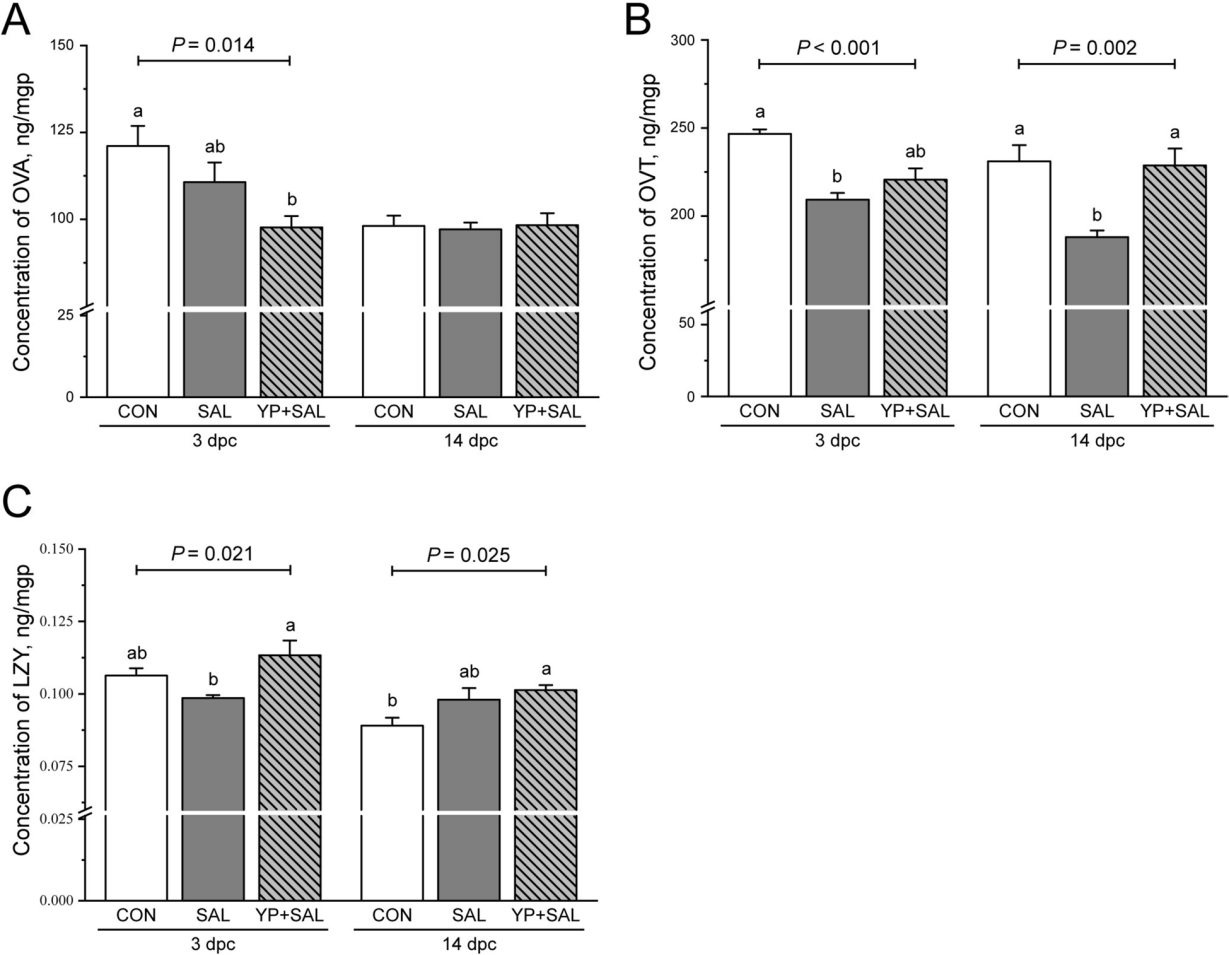
Effects of dietary yeast polysaccharides on oviduct mucosal levels of ovalbumin (**A**), ovotransferrin (**B**), and lysozyme (**C**) in laying hens challenged with *Salmonella* at 3 and 14 d post-challenge. CON, Unchallenged control (saline gavage); SAL, *Salmonella*-challenged group; YP + SAL, Yeast polysaccharides supplementation (1,000 mg/kg) with *Salmonella* challenge; OVA, Ovalbumin; OVT, Ovotransferrin; LYZ, Lysozyme; dpc, Days post-challenge. Data are represented with the means ± SE (*n* = 8). Bars with no common letters differ significantly (*P* < 0.05)

As shown in Fig. 4, at 3 d post-challenge, ovalbumin and ovotransferrin were reduced in challenged hens compared with CON (*P* < 0.05), although the magnitude of reduction differed between proteins. YP supplementation did not fully restore ovalbumin or ovotransferrin at this stage, but it prevented the decline in lysozyme, which remained comparable to or higher than CON (*P* < 0.05). By 14 d post-challenge, ovotransferrin remained suppressed in SAL hens, whereas YP supplementation alleviated this reduction (*P* < 0.01). Lysozyme displayed a distinct pattern, with YP-supplemented hens exhibiting the highest concentrations among all treatments at 14 d (*P* < 0.05).

#### Inflammatory cytokine concentrations in serum and oviduct mucosa

**Fig. 5:**
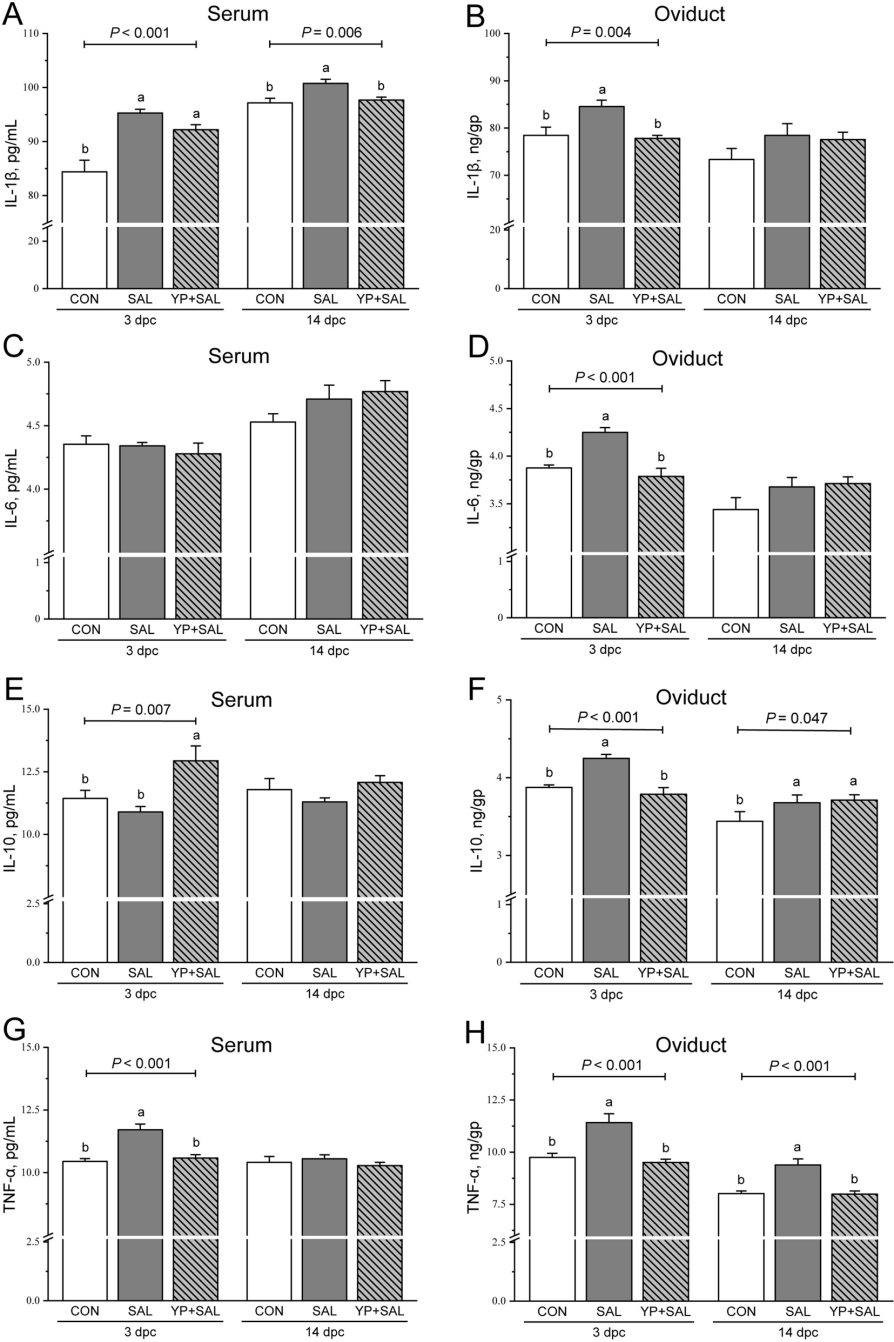
Effects of dietary yeast polysaccharides on serum (**A**, **C**, **E**, and **G**) and oviduct mucosal (**B**, **D**, **F**, and **H**) inflammatory cytokine levels of laying hens challenged with *Salmonella* at 3 and 14 d post-challenge. CON, Unchallenged control (saline gavage); SAL, *Salmonella*-challenged group; YP + SAL, Yeast polysaccharides supplementation (1,000 mg/kg) with *Salmonella* challenge; IL-1β, Interleukin-1 beta; IL-6, Interleukin-6; IL-10, Interleukin-10; TNF-α, Tumor necrosis factor-alpha; dpc, Days post-challenge. Data are represented with the means ± SE (*n* = 8). Bars with no common letters differ significantly (*P* < 0.05)

Serum and oviduct mucosal cytokine concentrations are presented in Fig. 5. At 3 d post-challenge, SAL hens showed higher serum IL-1β and TNF-α than CON (*P* < 0.05), whereas IL-6 remained unchanged (*P* > 0.05). Dietary YP supplementation elevated serum IL-10, with YP + SAL hens exhibiting higher IL-10 than both CON and SAL (*P* < 0.05). At 14 d post-challenge, only IL-1β remained increased in SAL hens, whereas levels in YP + SAL were comparable to those in CON (*P* < 0.05).

In the oviduct mucosa, SAL hens showed higher IL-1β, IL-6, IL-10, and TNF-α at 3 d post-challenge than CON (*P* < 0.05). These increases were alleviated by YP, resulting in cytokine levels in YP + SAL hens comparable to CON (*P* < 0.05). At 14 d post-challenge, mucosal IL-1β and IL-6 no longer differed among treatments (*P* > 0.05). However, SAL hens still showed elevated IL-10 and TNF-α compared with CON (*P* < 0.05), and only TNF-α was normalized by YP supplementation (*P* < 0.05).

#### Salmonella load and LPS levels

**Fig. 6:**
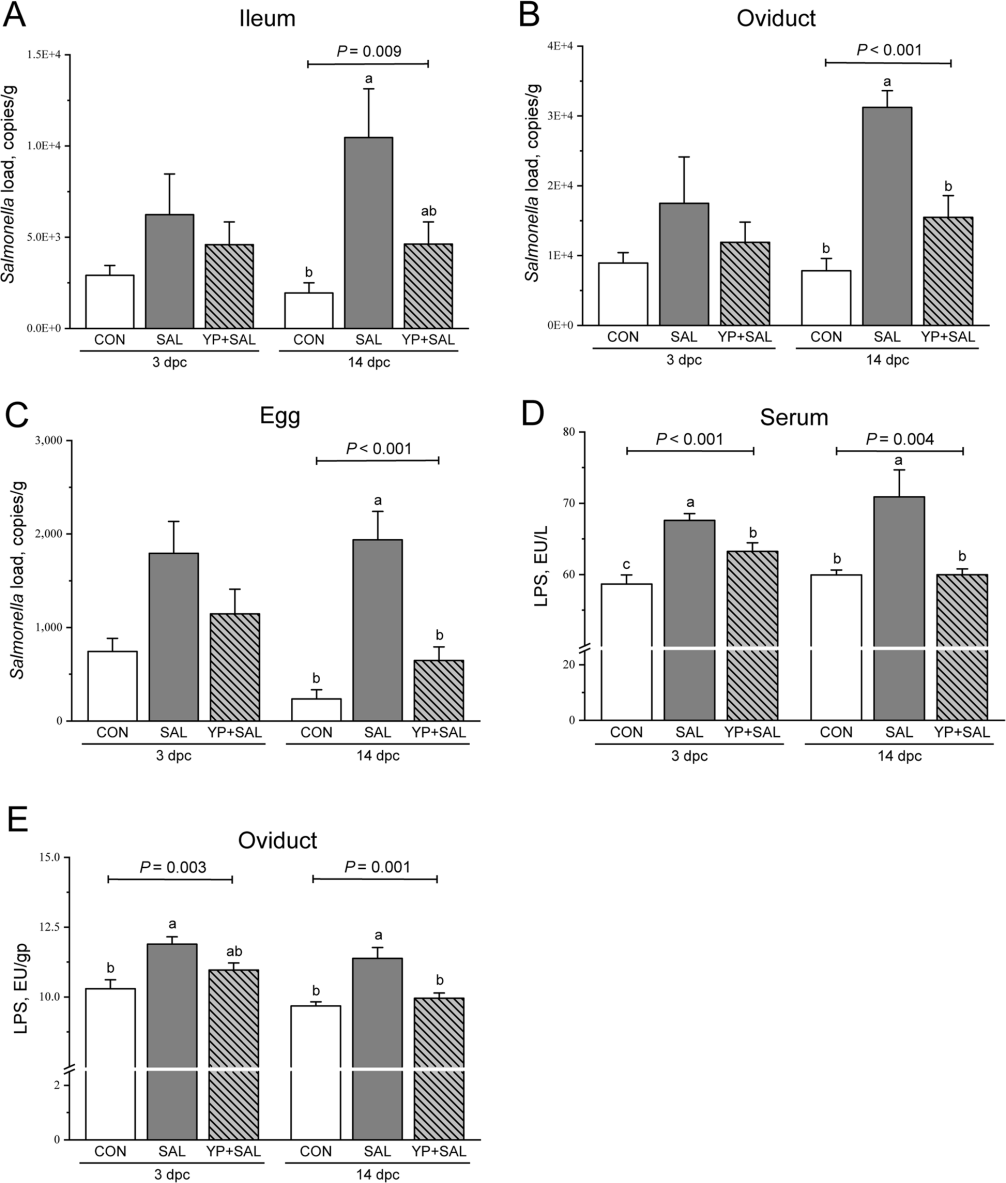
Effects of dietary yeast polysaccharides on *Salmonella* load and lipopolysaccharide levels in laying hens at 3 and 14 d post-challenge. CON, Unchallenged control (saline gavage); SAL, *Salmonella*-challenged group; YP + SAL, Yeast polysaccharides supplementation (1,000 mg/kg) with *Salmonella* challenge; LPS, Lipopolysaccharide; dpc, Days post-challenge. Data are represented with the means ± SE (*n* = 8). Bars with no common letters differ significantly (*P* < 0.05)

The *Salmonella* load and LPS levels are shown in Fig. 6. In the ileum, eggs, and oviduct, bacterial load did not differ among treatments at 3 d post-challenge (*P* > 0.05). By 14 d post-challenge, SAL hens had higher ileal *Salmonella* counts than CON, whereas YP + SAL hens showed intermediate values that remained lower than SAL (*P* < 0.05). A similar pattern was observed in the oviduct and eggs, whereas SAL hens showed the highest *Salmonella* load, while CON and YP + SAL hens had lower and comparable levels (*P* < 0.05).

Serum and oviduct LPS concentrations were elevated in SAL hens at both 3- and 14-d post-challenge (*P* < 0.05). Dietary YP supplementation mitigated this increase, with YP + SAL hens showing lower LPS levels than SAL and values comparable to CON at both time points (*P* < 0.05).

#### Diversity and composition of oviduct microbiota

**Fig. 7:**
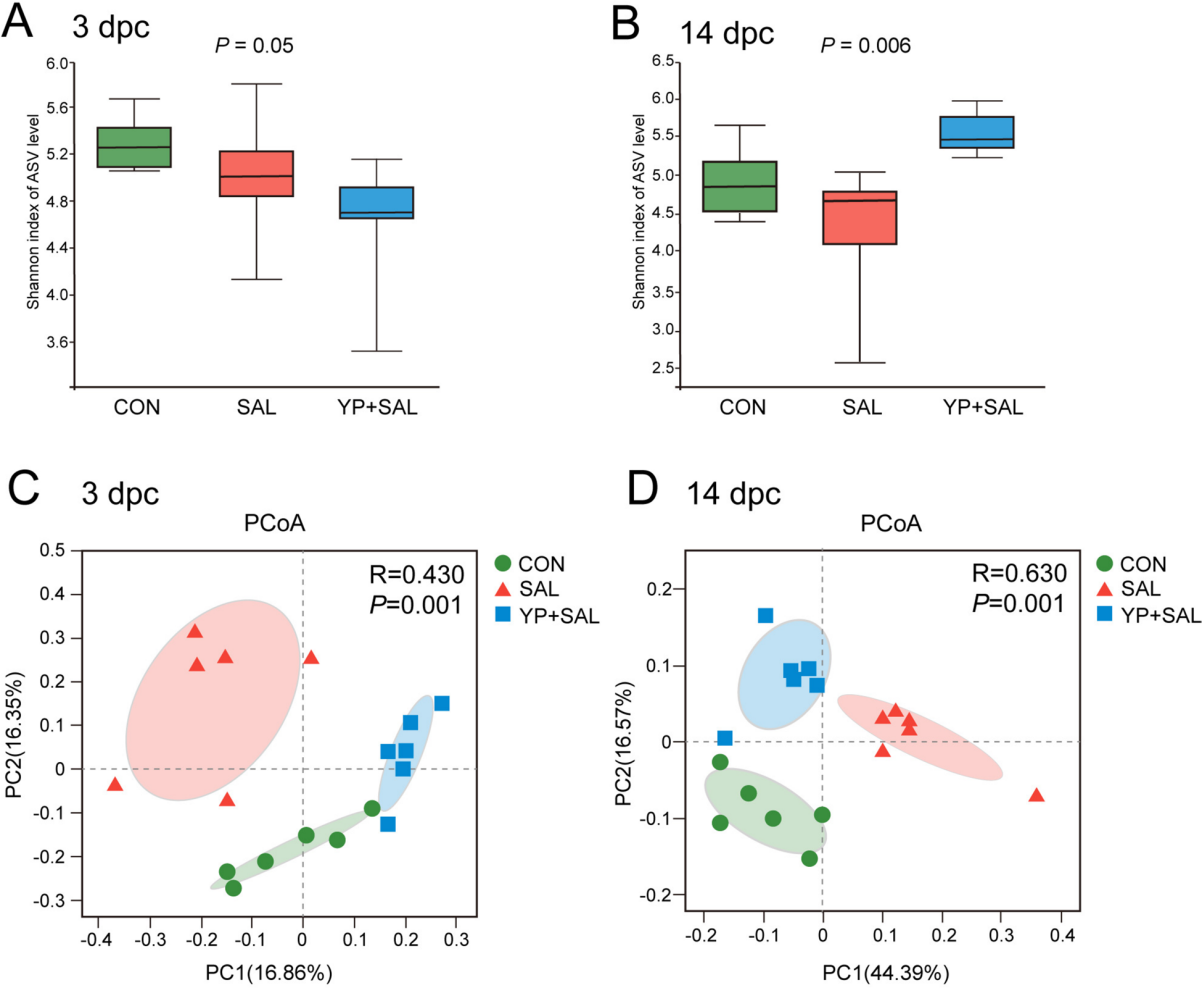
Effect of dietary yeast polysaccharides on oviduct bacterial diversity in laying hens challenged with *Salmonella* at 3 and 14 d post-challenge (*n* = 6). **A** and **B** Shannon evenness indices at 3 and 14 d post-challenge. **C** and **D** Principal coordinate analysis (PCoA) based on Bray–Curtis distances. Differences in microbial community structure among treatments were assessed using analysis of similarity. CON, Unchallenged control (saline gavage); SAL, *Salmonella*-challenged group; YP + SAL, Yeast polysaccharides supplementation (1,000 mg/kg) with *Salmonella* challenge; dpc, Days post-challenge

The alpha diversity of the oviduct microbiota was assessed using the Shannon index (Fig. 7A and B), with additional metrics including Chao1, ACE, and Simpson indices detailed in Table S2. At 3 d post-challenge, while the Shannon index showed a marginal trend (*P* = 0.05), the Simpson index indicated significant variation in community evenness (*P* < 0.05). By 14 d post-challenge, YP + SAL hens displayed the highest diversity, as reflected by significantly higher Shannon values (*P* < 0.05) and lower Simpson dominance scores (*P* < 0.05). Beta diversity was analyzed by PCoA based on Bray–Curtis distances (Fig. 7C and D). At 3 d post-challenge, the groups formed distinct clusters (R = 0.430, *P* = 0.001). This separation persisted at 14 d, with clear clustering of CON, SAL, and YP + SAL hens (R = 0.630, *P* = 0.001).

**Fig. 8:**
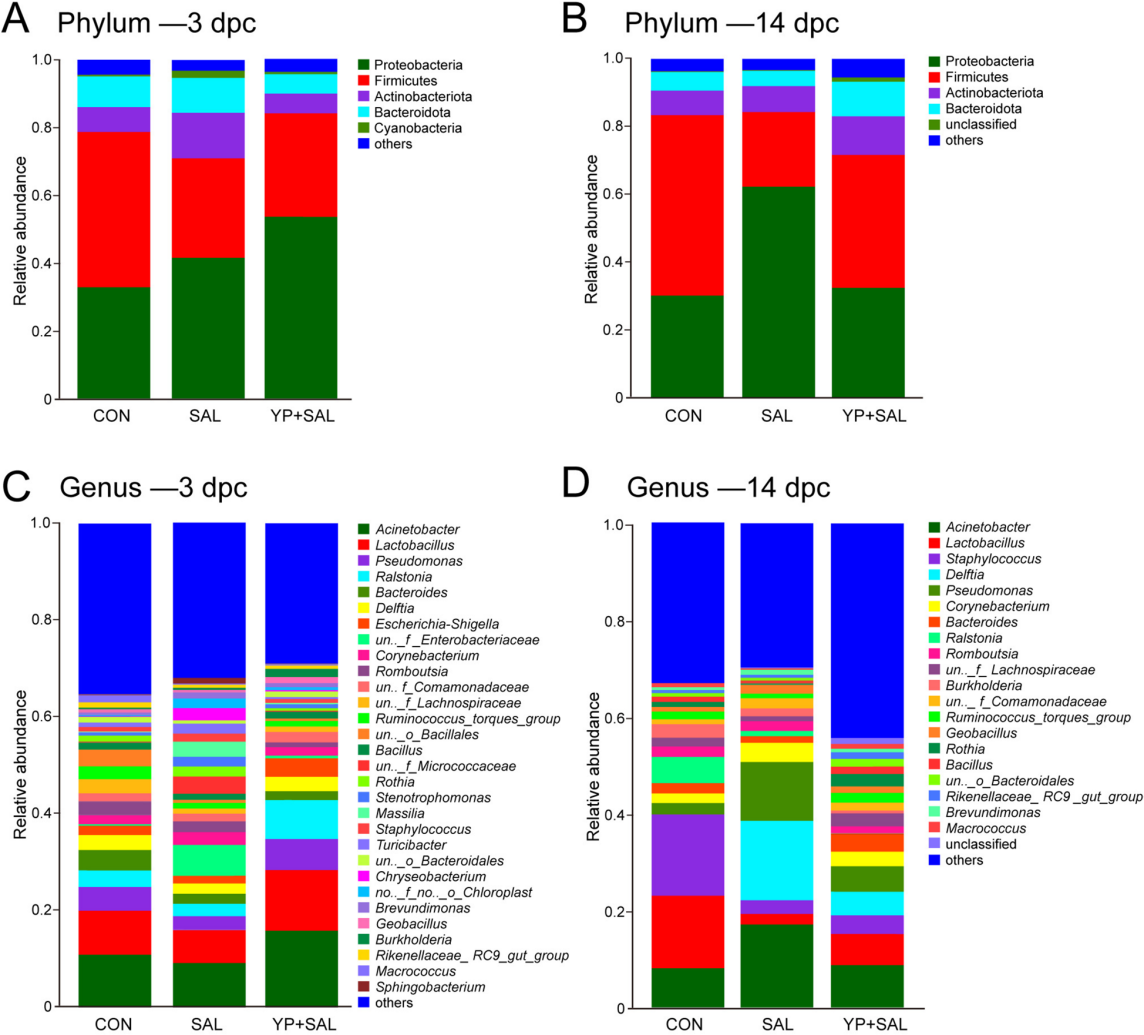
Effects of dietary yeast polysaccharides on oviduct bacterial composition at the phylum and genus levels in laying hens challenged with *Salmonella* at 3 and 14 d post-challenge (*n* = 6). CON, Unchallenged control (saline gavage); SAL, *Salmonella*-challenged group; YP + SAL, Yeast polysaccharides supplementation (1,000 mg/kg) with *Salmonella* challenge; dpc, Days post-challenge

Taxonomic composition of the oviduct microbiota was analyzed at the phylum and genus levels (Fig. 8). Across all treatments, the communities were dominated by Proteobacteria and Firmicutes, whereas Actinobacteriota, Bacteroidota, and other phyla accounted for smaller proportions. At 3 d post-challenge, *Salmonella* inoculation shifted the balance between the two dominant phyla, increasing the relative abundance of Proteobacteria and concomitantly reducing Firmicutes compared with CON. Supplementation with YP largely counteracted these changes, yielding a phylum-level profile in YP + SAL hens that was closer to that of CON. A similar pattern persisted at 14 d post-challenge, with Proteobacteria enrichment and Firmicutes depletion in SAL hens, and partial restoration of both phyla in the YP + SAL group. At the genus level, *Lactobacillus*, a key Firmicutes member, showed a consistent response over time: its relative abundance decreased in SAL hens at both 3 and 14 d post-challenge, whereas YP supplementation increased *Lactobacillus* compared with SAL and restored it to or above control levels. At 14 d post-challenge, *Salmonella* challenge also resulted in an increased abundance of *Acinetobacter*, which was reduced in YP + SAL hens toward control values, whereas *Staphylococcus* was depleted in both challenged groups, regardless of YP supplementation relative to CON.

**Fig. 9:**
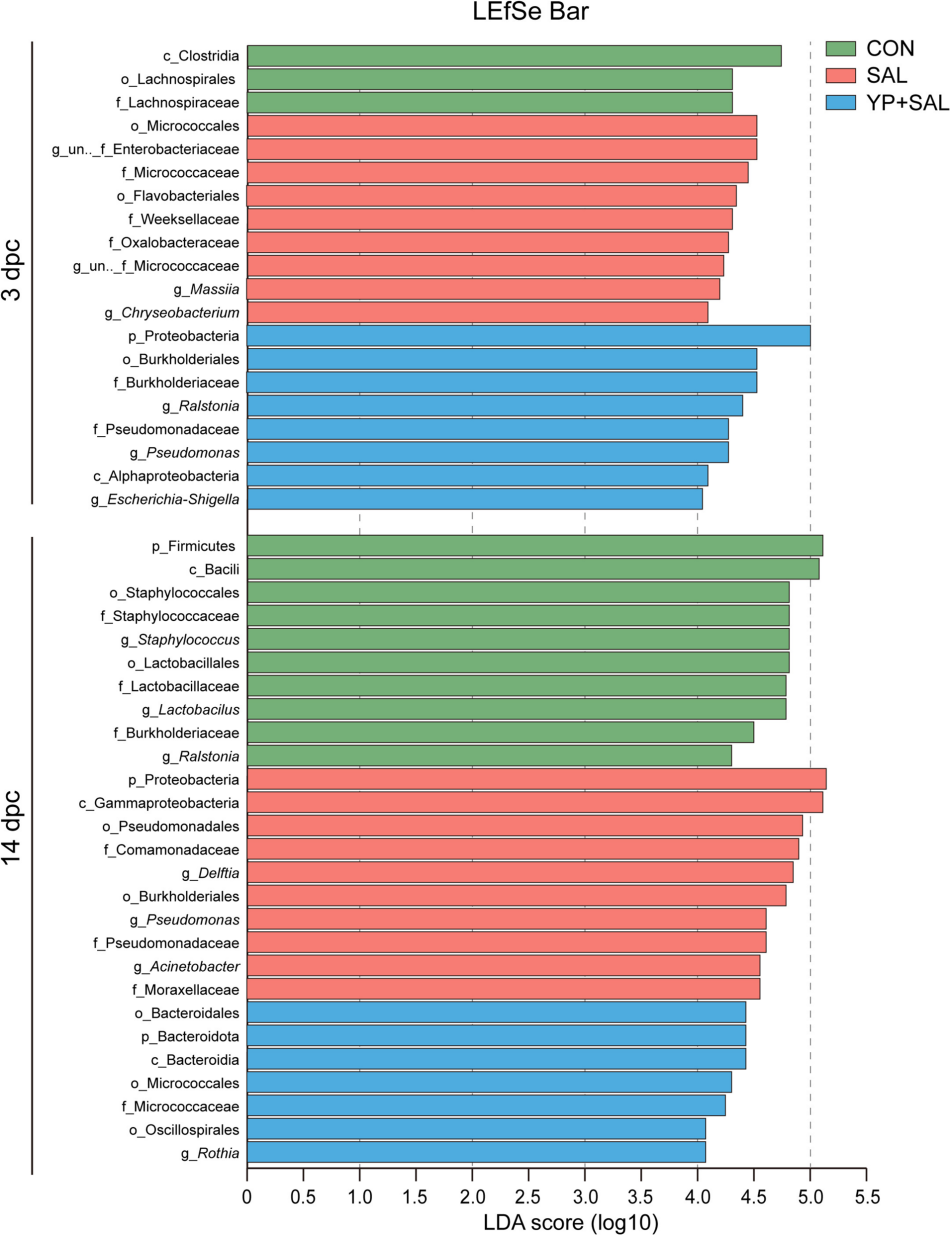
Linear discriminant analysis (LDA) scores of differentially abundant oviduct microbiota taxa identified by LEfSe (LDA > 4, *P* < 0.05). CON, Unchallenged control (saline gavage); SAL, *Salmonella*-challenged group; YP + SAL, Yeast polysaccharides supplementation (1,000 mg/kg) with *Salmonella* challenge; dpc, Days post-challenge

LEfSe analysis (LDA > 4, *P* < 0.05) was used to identify oviduct taxa that discriminated among treatments (Fig. 9). At 3 d post-challenge, the SAL group was characterized by enrichment of several Actinobacteriota and Bacteroidota lineages, including order Micrococcales (family Micrococcaceae and an unclassified genus within this family) and the Flavobacteriales–Weeksellaceae–*Chryseobacterium* cluster, together with Proteobacteria families Oxalobacteraceae (genus *Massilia*) and Enterobacteriaceae (unclassified genus). In contrast, YP + SAL hens were enriched for Proteobacteria-related taxa, notably the Burkholderiales–Burkholderiaceae–*Ralstonia* lineage and the family Pseudomonadaceae and its genus.

By 14 d post-challenge, SAL hens showed a distinct set of Proteobacteria biomarkers, including the phylum Proteobacteria, class Gammaproteobacteria, and its derivatives Pseudomonadales (families Pseudomonadaceae and Comamonadaceae; genera *Pseudomonas* and *Delftia*) and Moraxellaceae–*Acinetobacter*. In the YP + SAL group, discriminatory taxa shifted toward Bacteroidota and Actinobacteriota lineages, with enrichment of the Bacteroidota–Bacteroidia–Bacteroidales cluster and the Micrococcales–Micrococcaceae–*Rothia* lineage, as well as order Oscillospirales.

#### Correlation between oviduct microbiota and host parameters

**Fig. 10:**
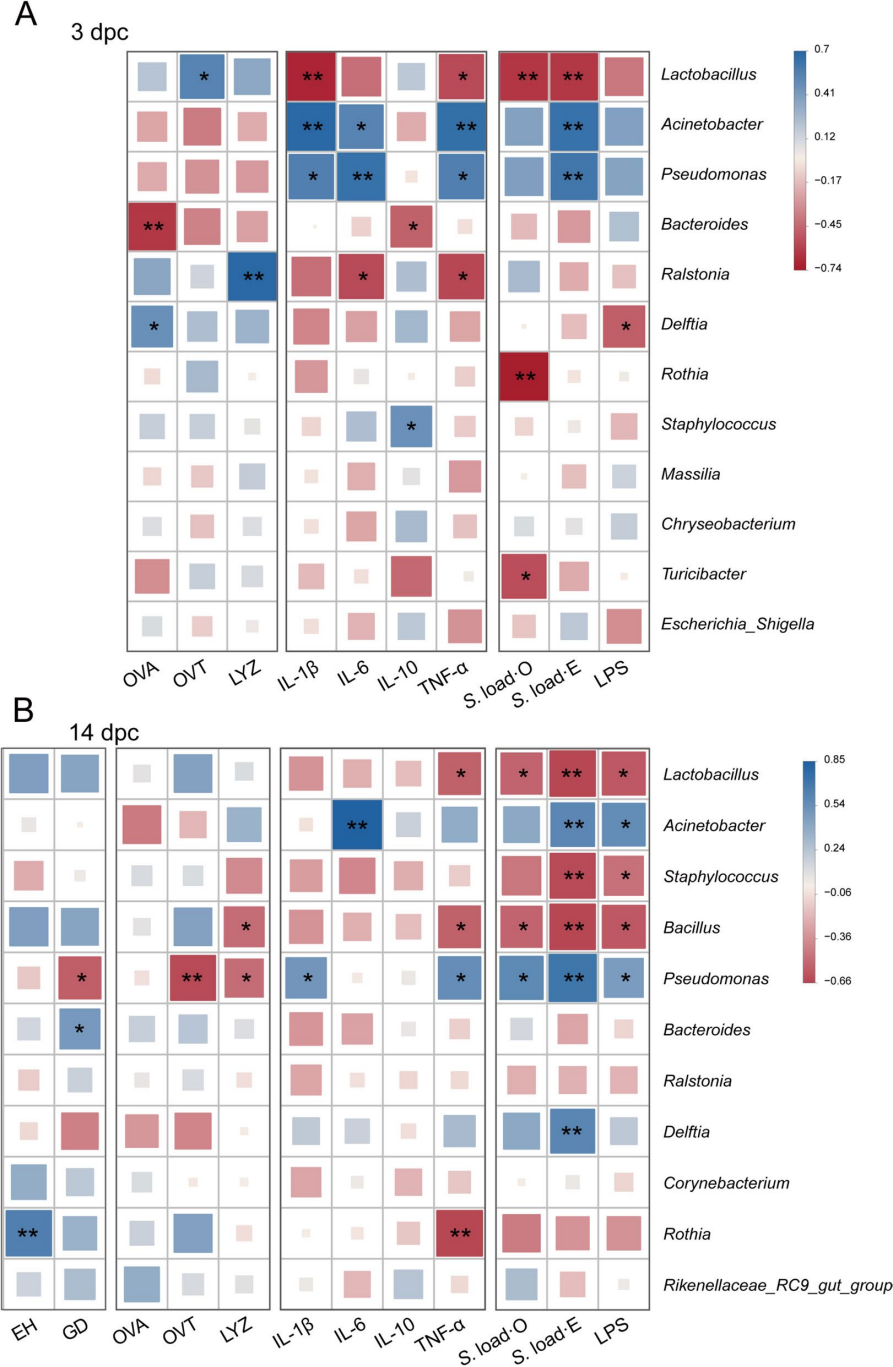
Spearman correlation analysis between differential oviductal bacterial genera and host phenotypic indices. **A** Correlation matrix at 3 d post-challenge (dpc). **B** Correlation matrix at 14 dpc. The columns represent host parameters including oviduct morphological indices, egg white proteins, inflammatory cytokines, and pathogen burdens; the rows represent the key differential bacterial genera. The color of the squares indicates the strength and direction of the Spearman correlation coefficient: blue represents a positive correlation, and red represents a negative correlation. Asterisks denote statistical significance (^*^P < 0.05, ^**^P < 0.01). EH, Epithelial height; GD, Glandular density; OVA, Ovalbumin; OVT, Ovotransferrin; LYZ, Lysozyme; S. load-O, *Salmonella* load in the oviduct; S. load-E, *Salmonella* load in eggs; LPS, Lipopolysaccharide

Spearman correlation analysis was performed to link the alterations in oviduct microbiota with host immune and barrier parameters (Fig. 10). At 3 d post-challenge, the genus *Lactobacillus* exhibited significant negative correlations with pro-inflammatory cytokines (IL-1β and TNF-α) and *Salmonella* loads in both the oviduct and eggs (*P* < 0.05). In contrast, *Acinetobacter* and *Pseudomonas* showed positive correlations with IL-6, TNF-α, and *Salmonella* loads in eggs (*P* < 0.05). At 14 d post-challenge, *Lactobacillus* maintained negative correlations with TNF-α, *Salmonella* loads, and LPS (*P* < 0.05). Conversely, *Pseudomonas* was positively correlated with IL-1β, TNF-α, and pathogen burdens (*P* < 0.05), while showing negative correlations with glandular density and antimicrobial proteins (OVT, LYZ; *P* < 0.05). Additionally, *Rothia* was positively correlated with epithelial height (*P* < 0.05) and negatively correlated with TNF-α (*P* < 0.01).

## Discussion

In continuation of our earlier work in which YP enhanced immune competence and intestinal microbiota in laying hens, we now further examined their roles in lipid metabolism under basal conditions and in oviduct protection during *S.* Pullorum challenge. Under normal physiological conditions, YP improved indices related to lipid utilization and yolk pigmentation and elicited distinct shifts in circulating lipid- and bile acid-related metabolites. Under infectious stress, the same dietary intervention alleviated *Salmonella*-induced deterioration of egg internal quality, preserved magnum epithelial integrity. Furthermore, YP supplementation sustained oviductal antimicrobial protein secretion, attenuated inflammatory responses, and reduced pathogen burdens, while partially restoring oviductal microbial diversity. Taken together, these findings suggest that YP improves lipid utilization under basal conditions and may enhance oviduct resilience to *Salmonella* challenge in laying hens.

Dietary YP primarily influenced traits linked to lipid deposition in the egg under basal conditions. After 12 weeks of feeding, yolk color scores increased in a dose-dependent manner, which was accompanied by a significant improvement in ether extract digestibility, particularly at 500 and 1,000 mg/kg YP. This improvement in fat digestibility is physiologically meaningful because the intestinal uptake of yolk pigments relies on mixed micelles composed of dietary lipids and bile acids [19, 25]. Thus, enhanced lipid utilization would be expected to increase the absorption of lipid-soluble xanthophylls, providing a coherent explanation for the deeper yolk color observed with YP supplementation. This interpretation is also consistent with our previous findings that similar YP inclusion levels improved overall feed efficiency [14].

Plasma metabolomics further supported the notion that YP enhances lipid digestion and transport. Hens receiving 1,000 mg/kg YP exhibited higher circulating levels of several lysoPC and lysoPE species, together with increased choline and taurochenodeoxycholic acid. LysoPC plays a central role in mixed micelle formation and promotes intestinal uptake of fatty acids and carotenoids [26, 27], which aligns well with the positive correlations we observed between these phospholipid derivatives and both ether extract digestibility and yolk pigmentation. The concurrent elevation of taurine and taurine-conjugated bile acids implies an expansion of the bile acid pool with enhanced emulsifying capacity [28]. These metabolite shifts may reflect enhanced intestinal solubilization and absorption of dietary lipids and lipid-soluble pigments. In addition, YP supplementation increased plasma riboflavin, nicotinamide, glycine, threonine, and azelaic acid while reducing PE(38:6). Riboflavin and nicotinamide are indispensable cofactors for mitochondrial and peroxisomal β-oxidation, and riboflavin deficiency is known to impair hepatic lipid metabolism and promote lipid deposition in poultry [29, 30]. Their elevated circulating concentrations, together with increases in amino acids linked to one-carbon metabolism and antioxidant defense, suggest a potential metabolic shift toward improved fatty acid oxidation and enhanced protection against lipid peroxidation. The reduction in PE(38:6), a highly polyunsaturated phosphatidylethanolamine species susceptible to oxidative modification [31], further suggests adjustments in membrane phospholipid remodeling that may accompany changes in oxidative metabolism. Indeed, the significant negative correlation between PE(38:6) and phenotypic traits supports the view that minimizing oxidation-prone lipids contributes to optimized lipid metabolism. While direct causality relationships cannot be established from metabolite associations alone, the overall metabolic profile, together with the observed improvements in fat digestibility and the metabolite–phenotype correlations, provides coherent evidence for enhanced lipid utilization at the systemic level.

After establishing that YP improved lipid handling and yolk pigmentation under basal conditions, a second major objective of this study was to determine whether these hens were better able to withstand a subsequent *S.* Pullorum challenge at the level of oviduct function. *S.* Pullorum and related serovars are well known to persist in the reproductive tract, damage the oviduct epithelium and compromise both production and egg quality in laying hens [2, 32]. In the present model, infection induced a transient deterioration in eggshell thickness and albumen height at 2–3 d post-challenge, followed by a more sustained depression of albumen quality (height and Haugh unit) at 13–14 d. The fact that the long-lasting defect was confined to internal freshness fits well with the view that reproductive tract disturbances, particularly at the magnum, often present as watery or thinned albumen rather than consistently severe shell defects [32–34]. Notably, hens pre-fed YP showed only mild reduction in albumen height and Haugh unit at 13–14 d, suggesting a partial preservation of oviduct function under *Salmonella* challenge.

Histological and biochemical analyses of the magnum provide a mechanistic link between this preserved internal quality and the condition of the reproductive tract. The magnum is the principal site of albumen synthesis and secretion, where tubular glands produce major albumen proteins [35, 36]. In SAL hens, magnum mucosa exhibited epithelial discontinuities, surface erosion and enlarged luminal spaces, features typical of inflammatory or toxic injury that are known to compromise secretory capacity [37, 38]. In contrast, YP + SAL hens showed a more continuous epithelial lining and fewer erosive lesions, indicating better structural preservation. Critically, this morphological protection was mirrored by preservation of oviduct-derived albumen antimicrobial proteins. *Salmonella* challenge reduced ovalbumin and ovotransferrin concentrations and tended to depress lysozyme in the oviduct mucosa at 3 d post-challenge, whereas YP prevented the decline in lysozyme at this stage and partially restored ovotransferrin by 14 d. Ovotransferrin and lysozyme are key antimicrobial components of egg albumen that restrict bacterial growth through iron sequestration and cell wall lysis, respectively, and their concentrations have been linked to the antimicrobial capacity of eggs [39–41]. Overall, these findings suggest that YP supplementation protects magnum epithelial structure and maintains the secretion of key innate defense proteins, which are important for limiting bacterial colonization of the egg.

These structural and functional benefits at the oviduct level were accompanied by a clear modulation of systemic and local inflammatory responses. As expected for an invasive *Salmonella* infection, SAL hens exhibited elevated serum IL-1β and TNF-α, together with marked increases in IL-1β, IL-6, IL-10 and TNF-α in the oviduct mucosa during the early post-challenge phase, consistent with a robust pro-inflammatory response in the reproductive tract [2, 42]. Dietary YP attenuated this response: YP + SAL hens showed higher circulating IL-10 at 3 d post-challenge and oviduct cytokine profiles that were largely comparable to controls, and by 14 d post-challenge IL-1β and TNF-α were normalized in the YP + SAL group while remaining elevated in SAL hens. These findings echo our previous work, which showed that YP dampened LPS-induced overexpression of pro-inflammatory cytokines and enhanced barrier-supporting mediators in laying hens [14], and are consistent with other studies in poultry where yeast-derived β-glucan and mannan fractions moderated inflammatory tone while supporting mucosal integrity [43, 44]. In this context, the elevation of circulating taurine-conjugated bile acids and taurine may be relevant to inflammatory regulation [45, 46], however, mechanistic signaling pathways were not directly tested in this study. Overall, YP confers functional protection to the oviduct under *Salmonella* challenge by preserving tissue architecture, sustaining the secretion of key antimicrobial albumen proteins and rebalancing systemic and local cytokine responses.

A plausible upstream explanation for these milder inflammatory and structural lesions is that YP reduced *Salmonella* colonization and LPS exposure in challenged hens. By 14 d post-challenge, SAL hens showed elevated *Salmonella* counts in the ileum, oviduct and eggs, consistent with the well-documented ability of invasive *S. Enteritidis* to colonize the ovary and oviduct and contaminate eggs via vertical transmission [1, 42]. In contrast, YP + SAL hens displayed lower *Salmonella* loads in these tissues and reduced egg contamination, indicating partial suppression of bacterial persistence along the intestinal–reproductive axis. Consistent with our previous findings that YP enhances ileal barrier function and immunity [14], the present data confirm that this pathogen-reducing effect extends to the reproductive tract. Serum and oviduct LPS concentrations followed a similar pattern, being consistently higher in SAL hens than in controls, whereas YP + SAL hens maintained LPS levels close to baseline. This agrees with previous reports in broilers and layers showing that mannan-rich yeast cell-wall preparations and related prebiotic products decrease cecal *S. Enteritidis* colonization and, in some cases, internal organ or ovarian invasion [43, 47, 48]. Proposed mechanisms include direct binding of mannose-sensitive fimbrial adhesins on *Salmonella*, competitive exclusion by beneficial microbes, and the reinforcement of barrier integrity. These actions collectively limit systemic endotoxin translocation [43, 44]. In this context, the lower *Salmonella* and LPS burdens in YP-supplemented hens likely contributed to the attenuated inflammatory response and helped protect oviduct structure and egg internal quality during *Salmonella* challenge.

The oviduct microbiota data add a microbial dimension to the protective effects of YP observed at the structural and immunological levels. In agreement with previous findings, the magnum microbial community was dominated by Proteobacteria, Firmicutes, Bacteroidota and Actinobacteriota, phyla typical of the hen reproductive tract [6, 7, 49]. Under *Salmonella* challenge, however, this balance was disturbed: SAL hens showed a shift toward Proteobacteria enrichment and Firmicutes depletion, together with a reduction in Shannon diversity at 14 d post-challenge, whereas YP + SAL hens maintained the highest diversity and a phylum-level profile closer to controls. This pattern is reminiscent of the broader concept that increased Proteobacteria and reduced diversity constitute a microbial signature of dysbiosis and mucosal dysfunction [50–52], and suggests that YP helped steer the oviduct microbiota away from a dysbiotic Proteobacteria-dominated configuration and toward a more resilient community structure.

Within this framework, the behavior of *Lactobacillus* is particularly noteworthy. Across time points, *Salmonella* challenge was associated with a decline in *Lactobacillus* abundance, whereas YP supplementation restored or even increased *Lactobacillus* relative to both SAL and CON. Recent studies have linked higher reproductive tract *Lactobacillus* levels with reduced oviduct inflammation in hens, and with improved oviduct barrier function when *Lactobacillus* strains are administered intravaginally [9, 10]. Evidence indicates that *Lactobacillus*-dominated reproductive microbiota is similarly associated with lower pathogen burden and improved mucosal health [53, 54]. These observations support the view that the *Lactobacillus*-enriched profile seen in YP + SAL hens represents a more functionally favorable state for the oviduct, a notion further supported by the significant negative correlations we observed between *Lactobacillus* abundance and both pro-inflammatory cytokines and *Salmonella* burdens. In contrast, several genera that contributed to the SAL-associated LEfSe signature have been linked to opportunistic or potential pathogenicity. SAL hens were characterized by Proteobacteria biomarkers including genera *Acinetobacte*r, *Pseudomonas*, and *Delftia*. Members of *Acinetobacter* and *Pseudomonas* are common colonizers of the poultry reproductive tract and egg surface and are frequently associated with spoilage or opportunistic infections [49, 55]. This opportunistic pathogenicity was corroborated by our correlation data, where these genera showed strong positive associations with mucosal inflammation and infection severity. By contrast, discriminatory taxa in the YP + SAL group shifted toward Bacteroidales and Actinobacteriota lineages such as *Rothia*, as well as Oscillospirales families, which are more commonly discussed as commensal or metabolically active members [56, 57]. Notably, *Rothia* was positively correlated with luminal epithelial height in our analysis, suggesting a potential contribution to mucosal structural integrity. Although functional roles of these specific taxa in the oviduct are not fully resolved, the overall pattern indicates that YP reduced the prominence of Proteobacteria-rich, opportunistic pathogens in favor of communities enriched in *Lactobacillus* and non-pathogenic commensals.

Together, these data show that YP supplementation was associated with a shift in oviduct microbiota composition toward higher diversity and relatively greater abundance of *Lactobacillus* with fewer Proteobacteria-enriched signatures in challenged hens. Such a shift may help reduce the burden of pathobionts and immunostimulatory LPS in the oviduct, potentially complementing the anti-inflammatory and barrier-preserving effects of YP and supporting the maintenance of egg internal quality under *Salmonella* challenge. While causal relationships between specific taxa and host outcomes require further validation, the present data position oviduct microbiota as a potential biological factor contributing to the reproductive tract resilience observed in YP-fed hens.

Several limitations of this study should be acknowledged. First, the *Salmonella* challenge was evaluated only at a single YP dose (1,000 mg/kg), and a dose–response relationship under infection stress was not established. Second, the observed shifts in oviduct microbiota are compositional and correlative. Functional validation studies, such as microbiota transplantation, targeted metabolites (SCFAs/bile acids), or culture-based assays are needed to definitively establish causality. Third, the post-challenge follow-up focused on short-term egg quality and tissue endpoints, and long-term production outcomes (e.g., laying performance, and longer-term egg safety) were not assessed and warrant future investigation.

## Conclusion

This study extends previous work on yeast polysaccharides in laying hens by demonstrating coordinated effects on lipid metabolism and oviduct defense. Collectively, YP improved lipid utilization under basal conditions and mitigated *Salmonella*-associated oviduct injury and egg internal quality deterioration, accompanied by reduced inflammatory and endotoxin burdens and a shift toward a more diverse, *Lactobacillus*-enriched oviduct microbiota. Overall, YP represents a promising nutritional candidate to support lipid utilization and reproductive tract resilience. Future studies should address the limitations of this work, particularly regarding long-term efficacy and functional verification of microbial interactions.

## Supplementary Information

Additional file 1: Table S1. Identified plasma metabolites satisfying the predefined quality-control and annotation criteria.

Additional file 2: Table S2. Effect of dietary yeast polysaccharides on oviduct microbial α-diversity of laying hens challenged with *Salmonella*.

## Data Availability

The datasets generated during and/or analyzed during the current study are available from the corresponding author on reasonable request.

## Abbreviations

ASV: Amplicon sequence variant
ATTD: Apparent total tract digestibility
BCA: Bicinchoninic acid
CFU: Colony-forming unit
IL-1β: Interleukin-1 beta
IL-6: Interleukin-6
IL-10: Interleukin-10
LDA: Linear discriminant analysis
LEfSe: Linear discriminant analysis effect size
LPS: Lipopolysaccharide
LysoPC: Lysophosphatidylcholine
LysoPE: Lysophosphatidylethanolamine
MS/MS: Tandem mass spectrometry
OPLS-DA: Orthogonal projections to latent structures discriminant analysis
PCA: Principal component analysis
PCoA: Principal coordinate analysis
SEM: Standard error of the mean
VIP: Variable importance in projection
YP: Yeast polysaccharides
