# Supplementary material for: Yeast polysaccharides modulate lipid metabolism and restore oviduct inflammatory and microbial homeostasis to support egg quality in Salmonella Pullorum-challenged hens
Source: J Anim Sci Biotechnol. 2026 Apr 8;17:61. doi: 10.1186/s40104-026-01377-z (PMC13059334; doi:10.1186/s40104-026-01377-z)
Supplement: Supplementary file 2 — Additional file 2: Table S2. Effect of dietary yeast polysaccharides on oviduct microbial α-diversity of laying hens challenged with Salmonella. [file 40104_2026_1377_MOESM2_ESM.docx]

| Item^2^ | Treatment | | | *P* value |
| --- | --- | --- | --- | --- |
|  | CON | SAL | YP+SAL |  |
| 3 d post-challenge |  |  |  |  |
| Ace | 818.34±87.01 | 790.99±245.78 | 758.88±303.28 | 0.864 |
| Chao 1 | 786.19±77.6 | 760.6±226.9 | 662.38±238.6 | 0.567 |
| Coverage | 0.95±0.01 | 0.95±0.02 | 0.95±0.02 | 0.947 |
| Shannon | 5.3±0.25^a^ | 5.01±0.55^ab^ | 4.61±0.57^b^ | 0.050 |
| Simpson | 0.01±0.00^b^ | 0.03±0.03^ab^ | 0.04±0.05^a^ | 0.024 |
| 14 d post-challenge |  |  |  |  |
| Ace | 968.77±226.54 | 776.47±313.12 | 1044.68±224.05 | 0.343 |
| Chao 1 | 959.42±226.25 | 770.45±310.71 | 1044.44±222.13 | 0.312 |
| Coverage | 0.99±0.01 | 0.99±0.01 | 0.99±0.01 | 0.692 |
| Shannon | 4.89±0.47^ab^ | 4.29±0.92^b^ | 5.56±0.29^a^ | 0.006 |
| Simpson | 0.04±0.03^ab^ | 0.08±0.06^a^ | 0.01±0.01^b^ | 0.006 |

**Table S2**. Effect of dietary yeast polysaccharides on oviduct microbial α-diversity of laying hens challenged with *Salmonella*^1^

^a, b^ Within a row, means with no common letters differ significantly (*P* < 0.05)

^1^ Means were calculated from 6 replicates per treatment.

^2^ CON Unchallenged control (saline gavage); SAL *Salmonella*-challenged group; YP+SAL Yeast polysaccharides supplementation (1,000 mg/kg) with *Salmonella* challenge.
